# Urodynamic changes before and after endoscopic valve ablation in boys diagnosed with the posterior urethral valve without chronic renal failure

**DOI:** 10.1186/s12894-022-01170-w

**Published:** 2023-01-06

**Authors:** Zhiqiang Mo, Minglei Li, Xianghui Xie, Ning Sun, Weiping Zhang, Jun Tian, Hongcheng Song

**Affiliations:** 1 Urology Department, Shunyi Maternal and Children’s Hospital of Beijing Children’s Hospital, Beijing, China; 2 Urology Department, Beijing Children’s Hospital, Capital Medical University, National Center for Children’s Health, Beijing, China

**Keywords:** Posterior urethral valve, Valve ablation, Urodynamic study, Bladder function, Bladder compliance

## Abstract

**Introduction:**

Current research on the posterior urethral valve (PUV) mainly focuses on the follow-up of bladder function after valve ablation. However, few studies exist on the changes in bladder function before and after valve ablation.

**Objectives:**

To investigate the urodynamic changes before and after PUV ablation and determine the effect of operation on bladder function, in patients.

**Materials and methods:**

The clinical records of 38 boys diagnosed with PUV and undergone urodynamic exams before and after valve ablation were retrospectively reviewed. In addition, differences in patients’ radiographic studies and urodynamic characteristics between pre- and post-operation were evaluated. Moreover, the urodynamic data was compared using the paired t-test and all the data was expressed as means ± SEM. Additionally, *p* values less than 0.05 were considered to be statistically significant.

**Results:**

All the patients were diagnosed with PUV and the follow-up period after operation ranged between 9 and 114 months. The urodynamic exams were performed about 6 months after operation. The results revealed that bladder compliance improved from 8.49 ± 4.73 to 13.31 ± 6.78 ml/cmH_2_O while the maximum detrusor pressure decreased from 95.18 ± 37.59 to 50.71 ± 21.71 cmH_2_O, after valve ablation. Additionally, there were significant differences in the pre- and post-operation values of bladder compliance and maximum detrusor pressure (*p *< 0.05). However, there were no significant differences in the pre- and post-operation values with regard to the residual urine volume, maximum bladder volume and maximum urinary flow rate (*p* > 0.05).

**Conclusions:**

The adequacy of the COPUM incision is necessary. But the study showed that endoscopic valve ablation couldn’t by itself completely improve the bladder function of patients diagnosed with PUV. However, it was able to improve bladder compliance and decrease maximum detrusor pressure to a certain extent. However, bladder compliance still couldn’t reach the normal level.

## Introduction

According to a previous report, the Posterior Urethral Valve (PUV) accounted for more than half of Lower Urinary Tract Obstruction (LUTO) in children [1]. It is a challenging medical condition in pediatric urology and affects one in every five to eight thousand newborn boys [2, 3]. Although congenital posterior urethral membrane (COPUM) is a local obstruction below the verumontanum, the anomaly also affects the bladder function. Unfortunately, the disorder is usually at a serious stage at the point of diagnosis. In addition, more than half of the patients diagnosed with PUV develop the End Stage Renal Disease (ESRD) even when the obstruction is cleared in the fetal or neonatal period [4]. Notably, valve ablation significantly improves the short-term survival rate and reduces symptoms. However, that this procedure alone how to change the long-term prognosis of this disorder has not been numerically documented previously [5]. Therefore, valve ablation is currently the basic method of treating COPUM. The extent to which a simple valve ablation can improve bladder function and alleviate upper urinary tract problems remains largely unclear. Although several comparative studies exist on the change in bladder function before and after urethral valve ablation, they lack a comprehensive description. For example, Krishna et al. reported their research results on the bladder function changes of 6 patients diagnosed with PUV [6] but lack of detailed characterization. Additional research by McKay et al. in 2019 also showed that deterioration of bladder function was an independent risk factor for ESRD [7]. The study highlighted the importance of maintaining good bladder function by studying patients diagnosed with PUV in childhood and eventually underwent renal transplantation due to ESRD in adolescence. Herein, the clinical data of 38 patients diagnosed with PUV and treated at the Beijing Children’s Hospital (BCH) between August 2013 and March 2022 were analyzed. All the patients underwent urodynamic examinations before and after operation. This was done to understand the effect of operation on bladder function.

## Materials and methods

### General information

Thirty-eight boys who had undergone valve ablation were included in the study. Their ages ranged 4 months to 9 years and 2 months with a mean age of 3 years and 5 months.

Prenatal ultrasound screening in 25 cases showed different degrees of bilateral hydronephrosis, which were not further treated in intrauterine except for observation.

Prior to valve ablation, bilateral bladder-ureter reimplantation had been performed on 3 patients in a separate hospital but had not yet been diagnosed as PUV. Additionally, 2 patients had been diagnosed with ‘Pyelo-ureteric Junction Obstruction’ (UPJO) from a different hospital. The patient had undergone pyeloplasty before admission in BCH.

### Clinical symptoms

The main clinical symptom in the patients (37/38) was obstructive urine flow characterized by weak and/or intermittent stream and short distance of urination. Among the 37 patients, 24 had slight difficulties in urinating, 11 had obvious obstructive urine flow while 2 had urinary dribbling and fever. Moreover, out of the 11 who had obvious obstructive urine flow, 10 patients’ parents had noticed the symptoms early and sought treatment without delay. The eleventh patient among the 11 with serious obstructive urine flow was diagnosed late because he was not taken to hospital on time.

Additionally, the left one patient among 38 had no obvious urinary symptoms previously although he was diagnosed with bilateral hydroureteronephrosis. This diagnosis was made by using ultrasonography techniques during examinations based on the abdominal pain experienced by the patient. It was however strange that obstructive urine flow occurred after the patient had undergone pyeloplasty.

### Diagnostic methods and findings

All the 38 patients underwent several examinations including the renal function test, urinary ultrasonography, urodynamic studies by Solar Blue (Netherlands) as well as Voiding Cystourethrography (VCUG) and diagnosis was confirmed by cystoscopy. Notably, urodynamic studies were performed on all the patients 1 week before valve ablation. Two days before the urodynamic examination, the patient went to the urodynamic examination room to get familiar with the environment, reduce the anxiety and establish a good relationship with the medical stuffs. During the examination day, most patients could generally better cooperate with the stuffs and complete the whole process through watching cartoons accompanied by parents. Of course, for some children who still could not cooperate, oral chloral hydrate was applied to sedate them before UDS. All patients in our group underwent urodynamic examination without accepting anesthesia. The examinations were carried out in accordance with the International Children’s Continence Society (ICCS) specification. Moreover, Lower Urinary Tract Obstruction (LUTO) was observed in all the 38 patients. Furthermore, urinary ultrasound revealed the presence of hydroureteronephrosis at varying levels of severity in all the 38 patients. In addition, the levels of urea, nitrogen and creatinine in the blood were normal in all the patients before valve ablation. The PUV was diagnosed through VCUG in 32 patients while 25 boys were diagnosed with Vesico-ureteral Reflux (VUR). It is noteworthy that one patient was suspected with both PUV and AUV through VCUG before operation.

### Treatment

All the 38 patients underwent cystoscopy and valve ablation under general anesthesia after complete preoperative preparation. During the operation, cystoscopes of appropriate sizes were selected to examine the urethra and bladder. In addition, the mucosa in all the 38 bladders with varying degrees of roughness and mucosa trabecular formation were examined. Careful examination of the urethra revealed that COPUM was present in the distal areas of the verumontanum in all the 38 patients.

Additionally, an incision was made at the 12 o’clock point of the valve using an electric scalpel or cold knife. Extra incisions were further made at the 5 and 7 o’clock points if bilateral residual valves were still obvious. No other mechanical obstructions such as AUV were found finally in other parts of the urethra in all the 38 patients. After withdrawing the cystoscopes, surgeons squeezed the patient’s bladder by pressing the abdomen to determine whether the urine stream was strong and satisfactory and ascertained that they were, indeed. The operation was finished once the result of urination was good.

The study protocol was approved by the Ethics Committees of BCH and written informed consent was obtained from the legal guardians of all the participants.

### Statistical analysis

The paired t-test was utilized to compare the main clinical parameters and all the data were expressed as means ± SEM. P values less than 0.05 (p < 0.05) were considered to be statistically significant.

## Results

**Fig. 1:**
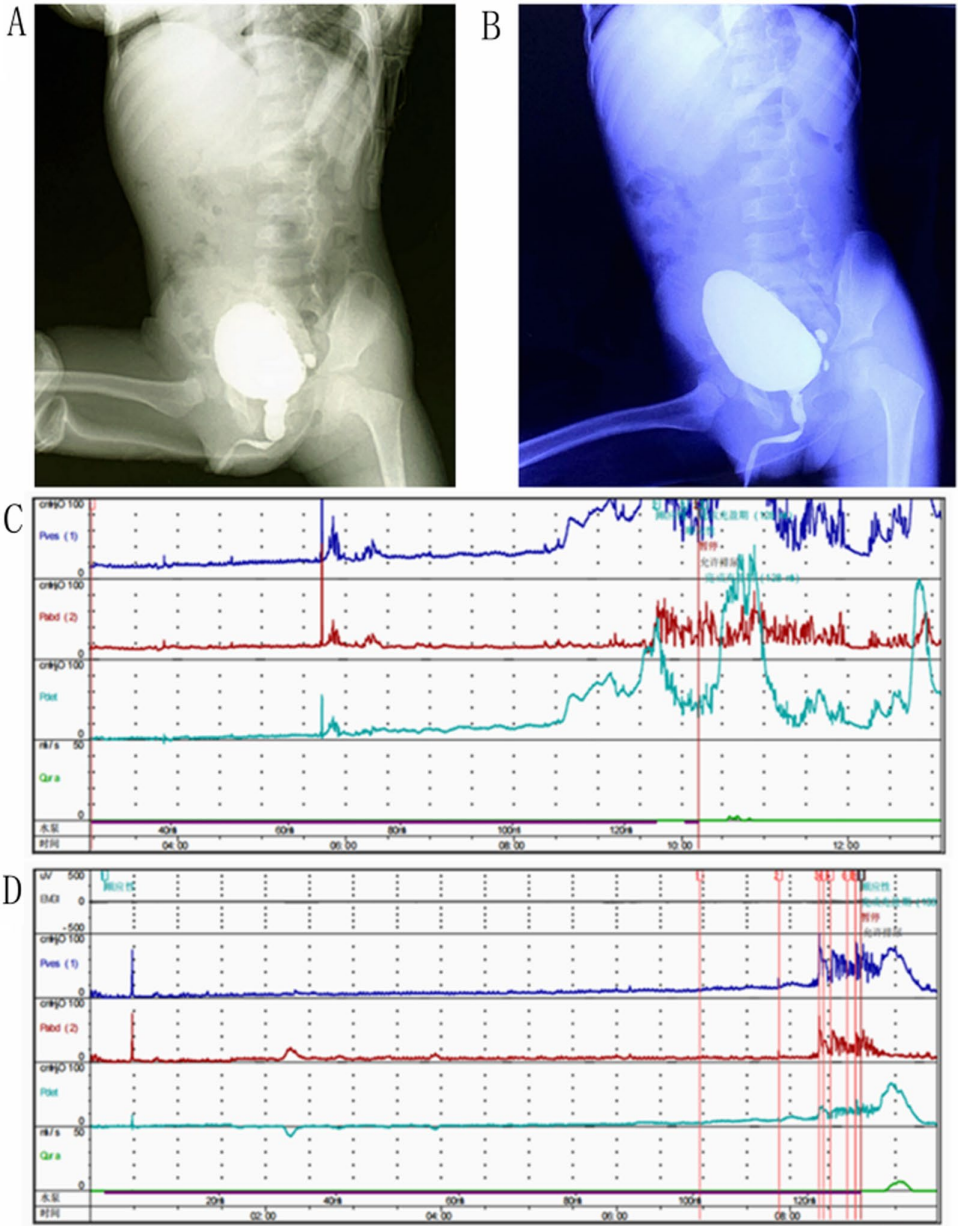
A 3-year old boy diagnosed with PUV with vesical diverticulum. **A** The VCUG of a patient highly suspected to have PUV before operation. **B** VCUG showed that LUTO disappeared 3 months after valve ablation. (**C**) Urodynamic studies revealed poor bladder compliance combined with extremely elevated detrusor pressure when voiding before valve ablation. (**D**) Urodynamic studies revealed improved bladder compliance combined with decreased detrusor pressure when voiding 3 months after valve ablation

**Fig. 2:**
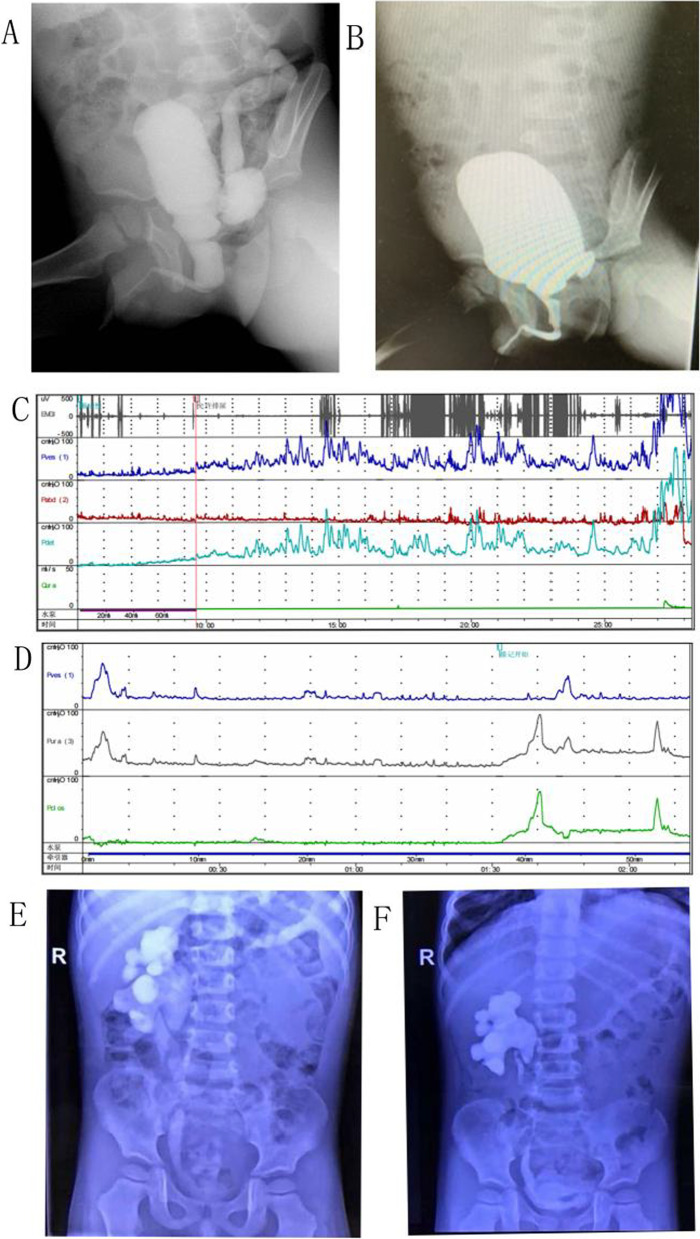
A 3-year old boy diagnosed with PUV and bilateral ureter hydronephrosis. He received bilateral ureters reimplantation 2 years before PUV ablation and his left renal function had heavily been damaged. **A** VCUG highly suspected PUV before operation, left VUR existed. **B** VCUG showed LUTO and VUR disappeared 3 months after operation. **C** Urodynamic studies revealed poor bladder compliance along with extremely elevated detrusor pressure when voiding before operation. **D** Urodynamic studies revealed improved bladder compliance along with decreased detrusor pressure (still higher than the normal) when voiding 3 months after operation. **E** IVP revealed the presence of right ureter hydronephrosis and no visualization on the left kidney before operation. **F** IVP revealed clearance of right ureter hydronephrosis and no visualization on the left kidney after operation

All the 38 patients were diagnosed with COPUM after which their situation during urination was observed. After the urethral catheters were removed, the 38 patients urinated spontaneously and smoothly in satisfactory volumes. Additionally, follow-up visits were made to all the patients for a period of between 9 and 114 months although the average follow-up time was 26.5 months. The follow-up was conducted both through telephone and reexamination at the clinic and neither deaths nor renal failure were reported during this period. It is worth mentioning that the urinary symptoms of 28 patients subsided or improved after operation although the symptoms in 9 patients did not disappear as much. Moreover, there was one patient whose symptoms were not obvious both before and after operation. Furthermore, the ultrasound findings after operation showed that hydroureteronephrosis decreased in 30 patients although there was no significant change in the other 8 boys. Post operation VCUG also showed that there was no urethral obstruction in all the 38 patients by direct vision analysis. Except for the qualitative evaluation, the quantitative value of the posterior urethra : anterior urethra ratio (PAR) was also used. PAR was computed by dividing maximum posterior urethral diameter by anterior urethral diameter. The postoperative PAR was 1.21 (1.19, 1.26) while the preoperative PAR was 4.21 (3.97, 4.63). This significant desent after operation indicates that valve ablation is successful. In addition, the VUR of 20 patients decreased or disappeared although the pre-operation degree of VUR in the other 5 patients remained at the same level even after operation. Urodynamic studies were also performed about 6 months after operation and the results before and after operation are compared in Table 1. Out of the 38 patients, 2 had pelvic floor muscle relaxation when urinating before operation while 15 others had pelvic floor muscle relaxation after operation. Therefore, the classic radiographic studies and urodynamic results of 2 patients are separately shown in Figs. 1 and 2.

**Table 1:** Comparison of bladder function between pre- and post-valve ablation

	Residual urine volume (ml)	Compliance (ml/cmH_2_O)	Maximum urinary flow rate (ml/s)	Maximum bladder volume (ml)	Pdet.Qmax (cmH_2_O)
Pre-	81.34 ± 86.31	8.49 ± 4.73	6.25 ± 3.72	149.39 ± 78.81	95.18 ± 37.59
Post-	64.18 ± 57.72	13.31 ± 6.78	8.01 ± 5.24	167.29 ± 74.15	50.71 ± 21.71
*p* value	0.094	0.001	0.057	0.150	0.003

*p* values less than 0.05 were considered to be statistically significant. There were significant differences in the pre- and post-operation values of compliance and Pdet.Qmax (*p* < 0.05)

Moreover, bladder compliance improved from 8.49 ± 4.73 to 13.31 ± 6.78 ml/cmH_2_O (*p* < 0.05) while the maximum detrusor pressure decreased from 95.18 ± 37.59 to 50.71 ± 21.71 cmH_2_O (*p* < 0.05 ), after valve ablation (Table 1). Notably, bladder compliance improved at varying extents in 35 patients but decreased in only 3 boys who also had unchanged symptoms and hydroureteronephrosis. In addition, the Pdet.Qmax of 37 patients declined after operation although 1 of them had a slightly higher Pdet.Qmax following surgery. On the contrary, the maximum bladder volume, the maximum urinary flow rate and the post voiding residual urine volume did not change significantly after valve ablation. Additionally, the maximum bladder volume changed from 149.39 ± 78.81 to 167.29 ± 74.15 ml while the post voiding residual urine volume changed from 81.34 ± 86.31 to 64.18 ± 57.72 ml (*p* > 0.05) after valve ablation. On the other hand, the maximum urinary flow rate changed from 6.25 ± 3.72 to 8.01 ± 5.24 ml/s (*p* > 0.05) after valve ablation. (Table 1). There were no significant differences between pre- and post-operation with regard to maximum bladder volumes, post-urinating residual urine volumes and maximum urinary flow rate.

## Discussion

Existing urodynamic literature on PUV focuses on the changes in urodynamic results and bladder function after valve ablation. In addition, only few studies exist that compare urodynamic data between pre- and post-valve ablation. The possible reason for the lack of such studies is that some doctors believe that valve surgery should be performed immediately after diagnosis, therefore leaving no time for urodynamics. However, it is possible to complete urodynamic examination after the diagnosis from VCUG then confirm the diagnosis through cystoscopy. This does not affect the diagnosis and treatment of patients and is conducive for a comprehensive assessment of the overall situation of patients before surgery. In this study, urodynamic studies were conducted about 6 months after operation. The results showed that bladder compliance improved while the maximum detrusor pressure decreased after valve ablation and these differences were significant between pre- and post-operation (*p* < 0.05). The decrease in maximal detrusor pressure was probably due to the relief of the mechanical obstruction in the posterior urethra and the reduced resistance which should have been overcome during urination. However, there were no significant differences in pre- and post-operation with regard to residual urine volume, maximum bladder volume and maximum urinary flow rate (*p* > 0.05). This therefore shows that not all the urodynamic parameters can improve significantly after valve ablation.

Krishna et al. [6], compared the urinary flow rate, bladder compliance and residual urine volume in 6 patients diagnosed with PUV. They conducted detailed urodynamic studies in all the 6 cases, 6 months after valve ablation. The results revealed that 3 of the 6 boys had normal upper tracts since they had a remarkable improvement in the peak urine flow rate after ablation and also displayed normal bladder pressure. However, the other 3 boys had hydroureteronephrosis and 2 had Chronic Renal Failure (CRF). Moreover, the boys had a markedly decreased functional bladder capacity with loss of compliance. They also had low bladder measurement volume and significant post voiding residual urine volume in spite of valve ablation, suggesting myogenic detrusor failure. In patients with long-standing obstruction but the upper tracts remained normal, the situation was associated with normal urodynamics. This was one of the few existing studies on urodynamic comparisons in patients with PUV before and after valve ablation.

In the present study, valve ablation cleared the obstruction anatomically (PAR < 2.2 after valve ablation [7]) although it was unable to completely improve bladder function. The results revealed that bladder compliance had indeed improved and detrusor pressure had also significantly decreased. However, there was no significant difference in bladder measurement volume, residual urine volume and maximum urinary flow rate. In addition, improvement in bladder compliance and decrease in detrusor pressure during urination did not appear perfectly in every patient. Interestingly, when the detrusor pressure significantly decreased during urination, there was an obvious improvement in bladder compliance. However, although bladder compliance improved statistically, it did not reach the development level among children of the same age. Values of P det at Qmax and BOOI in children are age independent and similar to those observed in adults [8]. Additionally, the bladder compliance of 3 cases who had no symptoms, improved while the detrusor pressure decreased significantly. In 1 case, the detrusor pressure increased, which may been the cause of the valve bladder syndrome and his bladder compliance was poor. Based on these findings, it is possible that a decrease in detrusor pressure may be one of the indicators useful in the prognosis of bladder function. Moreover, the changes in detrusor pressure are to some extent related to stage Chronic Kidney Disease (CKD) III B [1]. Furthermore, the patients in this study did not progress to ESRD probably because of their young age. Given that deterioration of renal function often occurs during adolescence [9], there was still a possibility of deterioration in renal function in the patients.

Simple valve ablation is the mainstream intervention for COPUM although doctors have tried a number of options including surgical treatment at the embryonic stage [10], urinary diversion [11] and bladder neck incision [12, 13], among others. Unfortunately, the overall prognosis was shown to be poor although these treatments led to reduced obstruction and obstructive urine flow [13]. In addition, many patients eventually progressed to the End-stage Renal Disease (ESRD) [14]. We are also trying some other personalized treatments, such as poor compliance of overactive detrusor with urodynamic force. We also use anticholinergic drugs as adjuvant therapy after surgery. In general, the operation is mainly to eliminate the obstruction made by COPUM and decrease detrusor pressure. In addition, alpha-blockers could be applied to further improve urination by targeting bladder neck relaxation. Individual treatment also includes circumcision to stream and to reduce related UTI. However, the prognosis of urethral valve is also related to many factors, including the age of onset, from large cities or rural areas, the severity of symptoms at the time of treatment, whether there is reflux, etc. These factors actually affect the severity of the disease at the time of treatment, but the pros and cons of these factors are still in disputes.

Additionally, patients with poor prognosis of PUV often have different degrees of urodynamic abnormalities [15]. Therefore, urodynamics has gained popularity in the diagnosis of PUV as well as during the follow-up of patients. It provides information on whether there is mechanical obstruction in the urethra and monitors changes in bladder function over time [4]. Moreover, an abnormal shape of the bladder is directly related to poor urodynamics [16]. Therefore, timely detection of functional changes before the appearance of organic lesions in the bladder can guide early treatment.

Furthermore, the major risks associated with PUV are the changes in bladder as well as renal function and an impairment of bladder function complicates the patient’s life [17]. Additionally, deterioration of bladder function accelerates the deterioration of renal function. Good bladder function delays the decline of and stabilizes renal function and even reverses the progression of disease. As such, it is important to highlight methods of improving and stabilizing bladder function. Although valve ablation solves the problem of persistent mechanical obstruction, it does not completely solve the functional challenges of bladder and upper urinary tract damage. It is therefore crucial to fully understand the effect of surgery on bladder function in order to reverse the deterioration of renal function as a result of PUV. Unfortunately, the number of patients in this study was small and a larger sample size should therefore be used in future research.

In addition to adjusting bladder function, PUV is often associated with VUR. Tourchi et al. [18] reported that the refluxes in patients diagnosed with PUV were relieved after the obstructions were cleared, consistent observations from the present study. Herein, refluxes in patients significantly decreased and even disappeared in some, after valve ablation and no further surgical treatment has been performed to date. However, patients with unrelieved as well as high-level refluxes and frequent urinary tract infections may require additional surgical treatment after careful evaluation. At the same time, oxybutynin may enhance the resolution of vesicoureteral refluxes and reduce hydronephrosis following endoscopic valve ablation in boys [19].

Most of the patients were diagnosed through prenatal ultrasound screening and a series of postnatal examinations, but some patients still didn’t have access to effective prenatal examinations to find urinary system abnormalities, but found them after the birth. The proportion of these patients in China is relatively large. Their main symptom is obstructive urine flow. Notably, although the common symptom for PUV is obstructive urine flow, some children do not display obvious clinical symptoms and there are no specific reasons for this. Additionally, patients showing mild or insignificant symptoms are often prone to missed diagnosis as well as delayed treatment and when they become older, they are diagnosed accidentally [20]. In this study, one case was more than 1 year at first diagnosis and treatment. He was diagnosed with bilateral hydronephrosis during his first visit to the hospital. It was however strange that obstructive urine flow occurred after the patient had undergone pyeloplasty. This may have been because when indwelling the catheter during the operation, the catheter pulled the valve which was originally attached to the urethral wall, to the obstructive state when the catheter went in and out of the urethra. This case mainly suggested that the possibility of PUV should be considered in the presence of bilateral upper urinary tract obstruction, even if there are no obvious clinical symptoms.

## Conclusion

The urodynamic study is an effective technique to monitor changes in the bladder condition of patients diagnosed with PUV. The adequacy of the COPUM incision is necessary. In addition, valve ablation does not completely restore bladder function. It is also does not lead to a significant increase in maximum bladder volume as well as the urinary flow rate and does not markedly decrease the residual urine volume. However, it improves bladder compliance and decreases maximum detrusor pressure to a certain extent. Moreover, the recovery of bladder compliance was more satisfactory when the maximum detrusor pressure decreased significantly. As such, PUV treatment based on valve ablation should be comprehensive.

## Data Availability

The datasets generated or analyzed during this study are available from the corresponding author on reasonable request.
